# The RNA Modification *N*^6^-methyladenosine and Its Implications in Human Disease

**DOI:** 10.1016/j.gpb.2017.03.002

**Published:** 2017-05-19

**Authors:** Pedro J. Batista

**Affiliations:** Laboratory of Cell Biology, Center for Cancer Research, National Cancer Institute, National Institutes of Health, Bethesda, MD 20892, USA

**Keywords:** *N*^6^-methyladenosine, Epitranscriptomics, Cancer, Viral replication, Metabolic disease

## Abstract

Impaired gene regulation lies at the heart of many disorders, including developmental diseases and **cancer**. Furthermore, the molecular pathways that control gene expression are often the target of cellular parasites, such as viruses. Gene expression is controlled through multiple mechanisms that are coordinated to ensure the proper and timely expression of each gene. Many of these mechanisms target the life cycle of the RNA molecule, from transcription to translation. Recently, another layer of regulation at the RNA level involving RNA modifications has gained renewed interest of the scientific community. The discovery that ***N*^6^-methyladenosine** (m^6^A), a modification present in mRNAs and long noncoding RNAs, can be removed by the activity of RNA demethylases, launched the field of **epitranscriptomics**; the study of how RNA function is regulated through the addition or removal of post-transcriptional modifications, similar to strategies used to regulate gene expression at the DNA and protein level. The abundance of RNA post-transcriptional modifications is determined by the activity of writer complexes (methylase) and eraser (RNA demethylase) proteins. Subsequently, the effects of RNA modifications materialize as changes in RNA structure and/or modulation of interactions between the modified RNA and RNA binding proteins or regulatory RNAs. Disruption of these pathways impairs gene expression and cellular function. This review focuses on the links between the RNA modification m^6^A and its implications in human diseases.

## The *N*^6^-methyladenosine RNA modification

The modification *N*^6^-methyladenosine (m^6^A) is present in several classes of RNAs, including the poly(A) fraction of mRNA, where it is the most abundant internal RNA modification [1]. m^6^A was first identified among poly(A) RNAs in the 1970s [2], [3], [4], [5], [6], [7], [8], [9]. In the poly(A) fraction of RNA, the m^6^A modification is present in a well-defined RNA motif, RRACH (R can be either A or G, and H can be A, C or U). The frequency of the methylation consensus motif far exceeds the m^6^A content of mRNA [1], suggesting that the primary sequence is likely necessary, but not sufficient for the m^6^A modification to occur. Indeed, high-throughput sequencing experiments have demonstrated that the m^6^A modification is not randomly distributed in mature transcripts, but is enriched at specific transcript landmarks such as the last transcribed exon, near the 5′ end, or in long exons [10], [11], [12], [13]. Furthermore, a correlation between m^6^A modification and tandem alternative polyadenylation (APA) site usage has been described. In a restricted group of mRNAs, knockdown of the m^6^A methylation complex, whose structure and function will be detailed below, resulted in a switch from proximal to distal APA site usage [10]. In a different study, sequencing of intact transcripts after m^6^A immunoprecipitation, revealed a strong bias toward the presence of m^6^A in transcript isoforms with shorter 3′UTRs [14], lacking regulatory elements.

The presence of m^6^A in the poly(A) fraction of RNA is widely conserved among eukaryotes [1]. In the yeast (*Saccharomyces cerevisiae*), cell fate decisions in response to starvation, such as dividing under a pseudo-hyphal foraging program or undergoing meiosis to form protective spores, are dependent on m^6^A [15], [16]. In *Arabidopsis thaliana,* loss of components of the methylation complex, and consequent loss of m^6^A, results in embryonic lethality [17], [18]. Later in development, loss of m^6^A yields plants with altered growth patterns and reduced apical dominance, with flowers that show defects in their floral organ number, size, and identity [19]. In the fruit fly (*Drosophila melanogaster*), loss of components of the methylase complex, and consequent loss of m^6^A, results in impaired neuronal function, flightless animals, and a sex bias toward maleness [20], [21]. In zebrafish (*Danio rerio*), the protein YTH *N*^6^-methyladenosine RNA binding protein 2 (Ythdf2), an m^6^A reader, facilitates removal of maternal mRNAs during the maternal-to-zygotic transition (MZT), an important developmental step. Loss of *ythdf2* decelerates the decay of m^6^A-modified maternal mRNAs and impedes zygotic genome activation [22]. Knockdown of components of the methylase complex in zebrafish embryos resulted in multiple developmental defects including smaller head and eyes, smaller brain ventricle, and curved notochord [23]. These observations suggest that in eukaryotes, m^6^A has crucial functions in development. In mice, genes encoding for methyltransferase like 3 (METTL3) and METTL14 are essential genes, and m^6^A is required for embryonic stem cells (ESC) to exit pluripotency [11], [24]. The landscape of m^6^A modification is very similar in mice and humans [11], [12]. Comparison of the m^6^A landscape of human, chimpanzee, and rhesus monkeys identified sets of m^6^A modification sites consistent with patterns of stabilizing or directional selection, with evolutionary gains of m^6^A signal correlating with evolution of higher mRNA levels [25].

## Writing the m^6^A modification: the methylase complex

**Figure 1:**
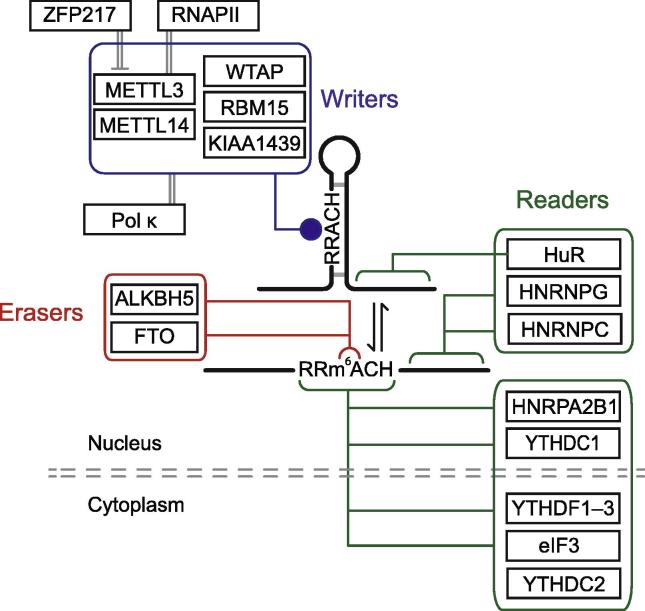
**The m^6^A regulatory pathway** Schematic representation of known components of the m^6^A regulatory pathways. The methylase complex (contained in the blue box) can modify adenosines in an RRACH context (R = G/A, H = G/A/C). The function of the methylase complex can be inhibited by ZNF217. The methylase complex interacts with RNAPII during transcription and recruits Pol κ to sites of DNA damage. Deposition of m^6^A can alter the RNA secondary structure. The demethylases (contained in the red box) can remove the methyl group. Two types of reader proteins (included in the green boxes) can interact with modified RNA through direct recognition of the methyl group or interaction with an RNA secondary structure induced by m^6^A modification. ALKBH5, alkB homolog 5; eIF3, eukaryotic initiation factor 3; FTO, fat mass and obesity-associated protein; HNRNPA2B1, heterogeneous nuclear ribonucleoprotein A2/B1; HuR, human antigen R; METTL3, methyltransferase like 3; Pol κ, DNA polymerase κ; PBM15, RNA binding motif protein 15; RNAPII, RNA polymerase II; WTAP, WT1-associated protein; YTHDC1, YTH domain containing 1; YTHDF, YTH *N*^6^-methyladenosine RNA binding protein; ZFP217, zinc finger protein 217.

The addition of the methyl group to the position *N*^6^ of adenosine is catalyzed by a large protein complex [26] (Figure 1). The core of this complex is a stable heterodimer of METTL3 [27] and METTL14 [28]. Although both proteins have methyltransferase domains, crystal structure studies of the heterodimer demonstrate that only METTL3 has catalytic activity, while METTL14 stabilizes METTL3 and its interaction with the RNA molecule [29], [30], [31]. A physical interaction between METTL3 and RNA polymerase II (RNAPII) has been observed under conditions that induce slow progression or frequent pausing of RNAPII, suggesting that m^6^A deposition occurs co-transcriptionally. Attenuated transcription leads to increased levels of m^6^A, which is detrimental to translation [32]. UV-induced DNA damage leads to increased levels of m^6^A and localization of METTL3 and METTL14 to sites of UV-induced DNA damage [33]. Accumulation of m^6^A at sites of UV-induced DNA damage requires ADP-ribose polymerase (PARP) and is required for early recruitment of DNA polymerase kappa (Pol κ), an enzyme implicated in both nucleotide excision repair and trans-lesion synthesis [33]. WT1-associated protein (WTAP) can interact with the METTL3/METTL14 heterodimer and is required for proper localization of the methylase complex to specific loci in the nucleus and efficient m^6^A modification [23], [28], [34], [35]. Additional proteins identified as interactors of METTL3/METTL14 include KIAA1429 [35] and RNA binding motif protein 15 (RBM15) [36]. While the molecular function of KIAA1429 remains unknown, RBM15, which is required for the function of X inactive specific transcript (*Xist*), has been shown to target the methylation complex to specific RRACH motifs to promote methylation [36]. The activity of the methylase complex can be inhibited by sequestration of METTL3 by the transcription factor zinc finger protein 217 (ZFP217), which in mouse ESCs (mESCs) is enriched at the promoters of m^6^A-modified RNAs [37].

## m^6^A is a dynamic mRNA modification: the demethylases

Discovery of RNA m^6^A demethylases raised the possibility that m^6^A can be dynamically regulated in the cell. Two enzymes capable of removing m^6^A have been identified to date: fat mass and obesity-associated protein (FTO) [38] and alkB homolog 5 (ALKBH5) [39], enzymes of the non-heme Fe(II)- and α-ketoglutarate-dependent dioxygenase AlkB family of proteins (Figure 1). While m^6^A methylation is broadly conserved across eukaryotes, orthologues of FTO and ALKBH5 have not been identified in *S. cerevisiae*, suggesting that in some organisms demethylation is passive [40]. *FTO* was first identified in genome-wide association studies and was linked to increased body mass index (BMI) [41], [42], [43]. FTO localizes to the nucleus, and in mice, mRNA levels of *Fto* are higher in the brain, particularly in the hypothalamic nuclei governing energy balance. Furthermore, *Fto* mRNA levels in the arcuate nucleus are regulated by feeding and fasting [44]. Initially, 3-methylthymine in single-stranded DNA (ssDNA) was proposed as the substrate of FTO [44]. A later study demonstrated that FTO preferred 3-methyluracil in single-stranded RNA (ssRNA) over 3-methylthymine in ssDNA [45]. Preference for a single-stranded substrate was confirmed by protein structure analysis [46]. In 2011, He and colleagues reported that FTO preferred m^6^A *in vitro*, and manipulation of *FTO in vivo* led to changes in cellular m^6^A levels [38]. Oxidative demethylation of m^6^A in RNA by FTO leads to the formation of *N*^6^-hydroxymethyladenosine and *N*^6^-formyladenosine, further expanding the range of chemical modifications present in RNAs [47]. Recently, FTO was described to show higher affinity for the modification *N*^6^,2′-*O*-dimethyladenosine (m^6^A_m_) as a substrate. m^6^A_m_ is found on the first nucleotide adjacent to the 7-methylguanosine cap. Transcripts with a m^6^A_m_ modification are more stable due to resistance to the mRNA-decapping enzyme, decapping mRNA 2 (DCP2) [48]. *Alkbh5* is expressed in most tissues, and is particularly abundant in the testes. While *Alkbh5* null mice are viable and appear anatomically normal, they exhibit impaired fertility due to the apoptosis of meiotic metaphase-stage spermatocytes [39]. A study of human asthenozoospermia patients found a correlation between elevated METTL3 and m^6^A levels, and sperm motility [49]. ALKBH5 localizes to the nucleus, and loss of ALKBH5 impairs mRNA export, RNA metabolism, and assembly of mRNA processing factors in nuclear speckles [39]. Structural studies of ALKBH5 revealed a region that excludes binding of dsDNA [50], [51], [52], [53]. Although the RRACH motif is critical for m^6^A methylation, it is not required for demethylase activity [54]. Rather, the conformational changes in the RNA structure induced by m^6^A modification provide a platform for interactions between the demethylases and the target RNA [54].

## The effect of m^6^A on RNA biology: the m^6^A readers

How the cell ‘perceives’ the presence of m^6^A on the RNA determines the downstream effect of this modification. The presence of m^6^A creates a binding site for proteins that preferentially recognize modified bases, and binding of m^6^A readers to target transcripts can determine multiple aspects of RNA metabolism. A number of proteins have been identified as direct m^6^A readers (Figure 1). Several proteins with a YTH domain were shown to bind m^6^A-modified RNAs. For instance, YTHDF2 selectively binds m^6^A-modified mRNAs, and recruits them to mRNA decaying sites, controlling mRNA stability [55]. YTHDF2 promotes RNA degradation through deadenylation, mediated by the CCR4-NOT complex [56]. YTHDF1, another m^6^A reader, interacts with the translation machinery and actively promotes translation of target mRNAs [57]. YTHDF3 cooperates with YTHDF1 to enhance translation, and impacts YTHDF2-mediated mRNA decay [58], [59]. Therefore, these three proteins, YTHDF1, 2, and 3, coordinate to provide spatiotemporal control over RNA metabolism. The protein YTH domain containing 1 (YTHDC1) is a nuclear m^6^A reader that regulates splicing and is required for the function of *Xist*, a long non-coding RNA (lncRNA) involved in X chromosome inactivation [36], [60]. The eukaryotic initiation factor 3 (eIF3) complex, through a multisubunit interface, interacts with m^6^A-modified 5′UTR to directly recruit the 40S preinitiation complex to the 5′UTR of mRNAs to stimulate translation initiation. This mechanism allows mRNA with m^6^A-modified 5′UTRs to be translated in a cap-independent way [61]. In the nucleus, heterogeneous nuclear ribonucleoprotein A2/B1 (HNRNPA2B1) binds m^6^A-modified transcripts and regulates splicing and microRNA (miRNA) maturation [62]. In addition, m^6^A has been shown to affect RNA secondary structure, modulating the ability of RNA binding proteins (RBPs) to bind to RNA in the neighborhood of m^6^A modification sites. This mechanism, termed “m^6^A-switch”, facilitates the binding of HNRNPC and HNRNPG to targets, to regulate mRNA abundance and splicing [63], [64], [65], [66], [67] (Figure 1). Binding of human antigen R (HuR) to RNA is constrained by distance to m^6^A modification sites, and is blocked by m^6^A on some transcripts [68].

## m^6^A and cancer

**Figure 2:**
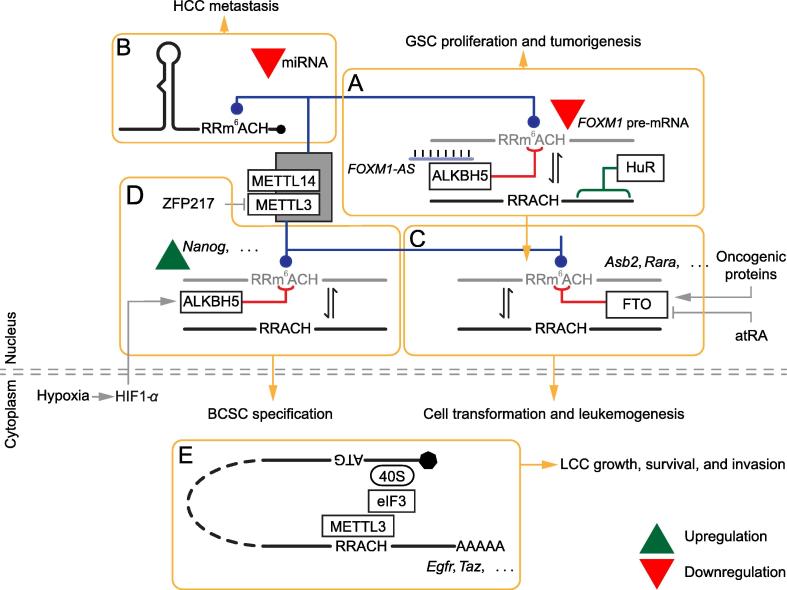
**Links between m^6^A regulation and cancer** Schematic representation of m^6^A pathway components and targets involved in cancer progression. Blue lines represent methylation, red lines represent demethylation and green lines indicate m^6^A reader proteins. **A.***FOXM1-AS* promotes interaction of ALKBH5 with *FOXM1*. HuR interacts with demethylated *FOXM1* pre-mRNA and enhances expression of *FOXM1*. **B.** Downregulation of METTL14 disrupts miRNA processing. **C.** Over-expression of FTO driven by leukemic oncogenic proteins leads to downregulation of target genes. **D.** Hypoxia leads to upregulation of ALKBH5 and ZFP217, resulting in loss of m^6^A levels in *NANOG* mRNA. **E.** METTL3 promotes translation of mRNA targets in the cytoplasm. ALKBH5, alkB homolog 5; ASB2, ankyrin repeat and SOCS box containing 2; atRA, all-*trans*-retinoic acid; BCSC, breast cancer stem cell; EGFR, epidermal growth factor receptor; eIF3, eukaryotic initiation factor 3; FOXM1, forkhead box M1; FOXM1-AS, FOXM1 antisense transcript; GSC, glioblastoma stem-like cell; HCC, hepatocellular carcinoma; HIF1α, hypoxia-inducible factor 1α; HuR, human antigen R; LCC, lung cancer cell; METTL3, methyltransferase like 3; RARA, retinoic acid receptor alpha; TAZ, tafazzin; ZFP217, zinc finger protein 217.

Studies on the role of METTL3 in ESCs uncovered several pluripotency transcription factors as targets of the m^6^A methylase complex, raising the possibility that pluripotency is regulated through m^6^A-dependent pathways. These studies demonstrated that loss of m^6^A leads to increased mRNA half-life of transcription factors such as *NANOG*, blocking the capacity of pluripotent cells to progress through differentiation [11], [24]. The phenotype observed on teratoma assays suggested that loss of m^6^A could also play a role in cancer progression [11], [24]. Indeed, multiple studies have implicated both m^6^A writers and erasers in cancer. Manipulation of m^6^A levels in glioblastoma stem-like cells (GSCs) severely impacted growth, self-renewal, and tumor development [69], [70]. Reduction of m^6^A levels, through knockdown *of Mettl3* and/or *Mettl14*, resulted in enhanced *in vitro* growth and self-renewal of GSCs and promoted the ability of GSCs to form brain tumors *in vivo*. At the molecular level, knockdown of *Mettl3* and/or *Mettl14* resulted in the upregulation of several oncogenes, including the genes encoding ADAM metallopeptidase domain 19 (ADAM19), EPH receptor A3 (EPHA3), and Kruppel-like factor 4 (KLF4), and downregulation of tumor suppressors such as genes encoding cyclin dependent kinase inhibitor 2A (CDKN2A), breast cancer 2, early onset (BRCA2), and tumor protein (TP53l11). Knockdown of *Adam19*, an m^6^A modified transcript, dramatically reduced growth and self-renewal of GSCs [69]. Increased levels of m^6^A in glioblastoma cells, achieved through overexpression of *Mettl3*, or treatment of cells with the FTO inhibitor ethyl ester form of meclofenamic acid (MA2), inhibited tumor progression [69]. High expression of ALKBH5 in glioblastoma patients predicts poor prognosis [70]. ALKBH5 is highly expressed in cell lines or patient-derived primary glioblastoma cultures enriched for GSCs, and knockdown of *ALKBH5* impairs GSC self-renewal *in vitro* and decreases proliferation and tumorigenesis of GSCs *in vivo*. Analysis of gene expression and m^6^A landscape of *ALKBH5* knockdown cells uncovered forkhead box M1 (FOXM1) as both a target of ALKBH5 and potential regulator of genes dysregulated upon *ALKBH5* knockdown. FOXM1 is a transcription factor with a critical role in the self-renewal and tumorigenesis of GSCs. Knockdown of *ALKBH5* leads to a gain of m^6^A on *FOXM1* nascent transcript and a decrease in association with HuR, resulting in a decrease of *FOXM1* expression. Interestingly, ALKBH5 interaction with *FOXM1* is promoted by *FOXM1-AS*, a lncRNA transcribed in the opposite orientation of *FOXM1*, with a 457-nt overlap at the 3′UTR (Figure 2A) [70]. These studies suggest that RNA demethylases are a potential target for anti-glioblastoma therapy [69], [70]. In hepatocellular carcinoma (HCC), downregulation of METTL14 results in lower m^6^A levels, and enhances the metastatic capacity of HCC cells [71]. The processing of the metastasis-associated miRNA miR126 requires METTL14 activity and is involved in HCC metastasis [71] (Figure 2B).

FTO was found to play an oncogenic role in acute myeloid leukemia (AML). *FTO* is expressed at higher levels in certain subtypes of AML, as it can be upregulated by leukemic oncogenes. Over-expression of *FTO* in two mixed-lineage leukemia (MLL)-rearranged AML cell lines promoted cell growth/proliferation and viability, while decreasing apoptosis and the global mRNA m^6^A level [72]. Notably, genes with m^6^A sites that are hypo-methylated upon overexpression of *FTO* tend to be downregulated in *FTO*-overexpressing AML cells. Overexpression of *FTO* leads to a reduction of m^6^A levels, downregulation of mRNA and protein levels in critical genes, such as those encoding ankyrin repeat and SOCS box containing 2 (ASB2) and retinoic acid receptor alpha (RARA). ASB2 and RARA are upregulated during normal hematopoiesis and are important regulators of all-*trans*-retinoic acid (atRA)-induced differentiation of leukemia cells. Demonstrating the importance of *ASB2* and *RARA* as FTO targets, the over-expression of these genes can rescue the effects of *FTO* over-expression, while knockdown was sufficient to rescue the inhibitory effect of *FTO* knockdown on AML cell growth and viability. Importantly, this study demonstrates that the inhibition of *ASB2* and *RARA* expression by FTO contributes to the response of AML cells to atRA treatment [72] (Figure 2C). Analysis of datasets from the Cancer Genome Atlas (TCGA) Research Network AML study revealed a strong association between mutations and/or copy number variation of m^6^A regulatory genes and *TP53* in AML patients. Furthermore, alterations in m^6^A regulatory genes confer a worse survival in AML patients [73]. In a meta-analysis study, the *FTO* polymorphism rs9939609 was found to be significantly associated with pancreatic cancer, although no other significant association was found [74].

Hypoxia is an important feature of the tumor microenvironment, and multiple studies have connected hypoxia to m^6^A regulatory pathways. The m^6^A demethylase ALKBH5 is a direct target of hypoxia-inducible factor 1α (HIF-1 α) [75]. In breast cancer cells, hypoxia leads to upregulation of ALKBH5, which then results in decreased m^6^A levels [76]. Upregulation of ALKBH5 also induced the loss of m^6^A in *NANOG* mRNA, which in turn increased its stability and NANOG protein levels in breast cancer stem cells (BCSCs). Therefore, hypoxia-dependent ALKBH5 upregulation mediates NANOG expression and BCSC specification and/or maintenance [76]. Exposure of breast cancer cells to hypoxia also leads to the upregulation of ZNF217, a transcription factor known to interact with METTL3 and inhibit its function [77] (Figure 2D). In gynecological tumor cell lines, hypoxia leads to a reduction of YTHDC1 protein levels, through changes in splicing that generate mRNA isoforms targeted by nonsense-mediated decay [78]. The m^6^A reader YTHDC2 [79] has been shown to promote translation of HIF-1 α and contributes to colon tumor metastasis [80]. These studies reveal how the tumor microenvironment can impact gene expression through the epitranscriptome, with devastating outcomes.

METTL3 has also been proposed to promote growth, survival, and invasive capacity of lung and cervical cancer cells in a methylation-independent manner [81]. Lin and colleagues described how METTL3 promotes the translation of specific mRNAs, including those encoding epidermal growth factor receptor (EGFR) and tafazzin (TAZ), through interaction with RNA, the translation initiation machinery, and the ribosome [81]. In this study the authors show that although *METTL3* knockdown has little effect on the steady state level of methylated mRNAs, the protein expression level of a specific group of genes was reduced. While knockdown of *METTL3* resulted in the re-localization of the *EGFR* and *TAZ* mRNAs to sub-polysome fractions, tethering of METTL3 to target mRNAs robustly enhanced translation. The catalytic activity of METTL3 is dispensable, as tethering a catalytically-dead METTL3 also enhances translation of target mRNA (Figure 2E). This study, with results in line with those of other studies concerning viruses that replicate in the cytoplasm (see below), uncovers a function for METTL3 in the cytoplasm.

## m^6^A and viral replication

**Figure 3:**
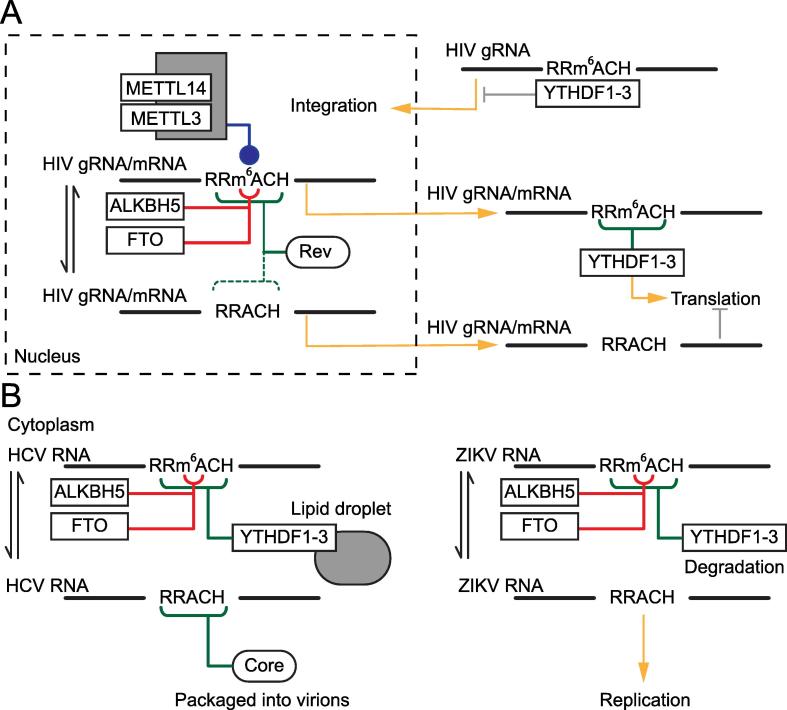
**m^6^A is involved in viral replication** Schematic representation of the intersection between m^6^A pathway and viral replication. Blue lines represent methylation, red lines represent demethylation and green lines indicate m^6^A reader proteins. **A.** Writers, erasers, and readers in the m^6^A pathway influence HIV RNA transport, stability, and translation, in both nucleus and cytoplasm. **B.** m^6^A pathway regulates viruses that replicate in the cytoplasm. ALKBH5, alkB homolog 5; FTO, fat mass and obesity-associated protein; HCV, hepatitis C virus; HIV, human immunodeficiency virus; METTL3, methyltransferase like 3; YTHDF, YTH *N*^6^-methyladenosine RNA binding protein; ZIKV, Zika virus.

The presence of m^6^A, and in some cases the exact location of the m^6^A modification, was observed in viral transcripts as early as the 1970’s. Pioneering work revealed the presence of m^6^A in Rous sarcoma virus (RSV), adenovirus type 2, Simian virus 40 (SV40), and influenza RNAs [82], [83], [84], [85], [86], [87], [88]. While the presence of m^6^A in viral RNAs was well established, the role of the modification in viral biology remained unknown. In an early attempt to understand how m^6^A influences RSV biology, the Beemon Lab mutated the consensus motif around methylated adenosines. Although these studies demonstrated that mutations of the RRACH motif resulted in loss of methylation, no effect on viral RNA processing or viral life cycle was found when the virus was modified to prevent m^6^A methylation [89], [90]. A different study, in which the researchers used a competitive inhibitor of methionine transferase, cycloleucine, suggested that m^6^A is required for the formation of late SV40 RNAs in untransformed cells [91], however it is important to note that cycloleucine is not a m^6^A-specific inhibitor, and the results should be interpreted with caution. Recently, work from three separate groups demonstrated that m^6^A plays an important role in HIV-1 biology. Lichinchi and colleagues [92] showed that acute viral infection triggers a massive increase in host and viral levels of m^6^A, and identified 56 host genes with a role in viral infection that are specifically m^6^A modified upon infection. The authors also demonstrate that loss of m^6^A decreased viral replication, while elevation of m^6^A levels through knockdown of *ALKBH5* resulted in increased replication of HIV. The authors focused on two m^6^A sites at the stem loop II (important structural motif) region of HIV-1 Rev response element (RRE), and demonstrated that one of the m^6^A sites, A7883 (in the loop region), is required to enhance binding of HIV-1 Rev protein to this structural RNA element, an interaction required for viral RNA to exit from the nucleus. Interestingly, A7883 is well conserved across isolates, suggesting the importance of this site. Further studies will be required to understand if Rev is a direct or indirect reader of m^6^A. Kennedy and colleagues also detected m^6^A, and showed that it is required for HIV-1 replication. The authors showed that the YTHDF1−3 proteins bind to and enhance expression of viral RNAs. Cullen and colleagues also demonstrated that four m^6^A-modified sites are conserved across isolates, again suggesting that maintaining m^6^A sites confers survival benefits [93]. Tirumuru and colleagues showed that the m^6^A reader proteins YTHDF1−3 bind HIV-1 RNA and inhibit HIV-1 post-entry infection by blocking viral reverse transcription and promoting degradation of the *gag* mRNA. At a later stage, m^6^A is required for production of Gag protein in virus producing cells, as m^6^A might promote translation of viral proteins in infected cells [94]. While there are some conflicting results between these studies, these papers demonstrate the importance of m^6^A on HIV-1 biology, and suggest that targeting this pathway might offer new tools to control viral infection (Figure 3A).

Viruses of the Flaviviridae family, which replicate exclusively in the cytoplasm, have been shown to be m^6^A modified [95], [96]. Comparison of m^6^A profiles across multiple Flaviviridae viruses suggests that the m^6^A modification is potentially conserved in this family [96]. Furthermore, m^6^A is conserved across multiple isolates of Zika virus (ZIKV) [95]. Loss of m^6^A or impairment of m^6^A reader proteins of the YTH family result in higher viral particle production of both ZIKV and hepatitis C virus (HCV), while loss of demethylase activity negatively impacts viral particle production. As observed with HIV infection, ZIKV infection leads to changes in the m^6^A profile of the host cell. In the case of ZIKV infection, new m^6^A peaks in host transcripts are enriched at the 5′UTR and the CDS, while the lost m^6^A peaks tend to localize at the exon junction and at the 3′UTR [95]. HCV infection leads to a re-localization of YTH proteins to loci of HCV particle production. YTH proteins interact with the HCV RNA and suppress viral production [96]. Mutagenesis studies on m^6^A sites in the viral *E1* gene of HCV suggest that the interaction of the viral RNA with host YTHDF proteins or viral core proteins is regulated by the presence of m^6^A on the viral gene [96] (Figure 3B).

## m^6^A and metabolic diseases

In mammals, metabolism, as well as other features of physiology, is regulated in a temporal way by the circadian system. At the cellular level, the circadian clock is ‘paced’ by feedback loops at the transcriptional and translational levels that result in periodic gene expression. Modification of RNA with m^6^A, through its effects on RNA metabolism, directly impacts the length of the circadian period, with loss of m^6^A resulting in circadian period elongation [97]. Metabolism, at the organism level, has also been linked to RNA post-transcriptional modifications through FTO. *FTO* was first associated with obesity in a series of population studies that linked variations in the first intron of *FTO* to elevated BMI [41], [42], [43]. Although the link between the *FTO* polymorphism and BMI has been confirmed in multiple studies, how expression of FTO leads to obesity is not well understood. Several studies propose that the polymorphisms in the first intron of *FTO* control expression of other genes, such as those encoding RPGRIP1 like (RPGRIP1L), iroquois-related homeobox 3 (IRX3), and IRX5 [98], [99], [100], [101]. Studies in mouse models, on both loss of function and overexpression of *Fto*, revealed a role for FTO in energy homeostasis and body composition [102], [103], [104]. Global loss of *Fto* resulted in postnatal growth retardation and significant reduction in adipose tissue and lean body mass [102]. A dominant missense point mutation (I367F) in the *Fto* gene resulted in reduced fat mass, increased energy expenditure, and unchanged physical activity without postnatal growth retardation or perinatal lethality [103]. Ubiquitous overexpression of *FTO* results in a dose-dependent increase in body and fat mass of mice [104]. Furthermore, FTO plays a critical role in the regulation of adipogenesis through its effect on RNA splicing, particularly for the gene encoding the adipogenic regulatory factor RUNX1 translocation partner 1 (RUNX1T1) [105]. In skeletal muscle cells, FTO-dependent demethylation of mRNA m^6^A methylation is involved in the regulation of skeletal muscle lipid accumulation [106]. In humans, a missense mutation in the coding region of *FTO* (R316Q), inactivates enzymatic function and results in a rare autosomal-recessive lethal syndrome [107].

## Concluding remarks

Organisms face innumerable challenges to their physiology, both during and after embryonic development. At the cellular level, the ability to execute developmental programs and respond to environmental challenges requires precise and coordinated control of gene expression. As a central player in gene expression, the RNA molecule is the target of several of these regulatory pathways, and disruption of any of these mechanisms can result in disease and have catastrophic consequences. This review focused on the link between pathways that rely on m^6^A to modulate gene expression and its implications in human disease. RNA post-transcriptional modifications, through their impact on transcription, splicing, mRNA stability, and rate of translation, regulate essential features of a cell. It is not surprising that dysregulation of these pathways is implicated in human disease. Although some details are still lacking, it is clear that the balance between methylation and demethylation at specific RNA transcripts plays a critical role in human health and represents an attractive target for therapy. Inhibition of the RNA demethylases ALKBH5 and FTO is a potential strategy to target cancer stem cells [69], [70], [76]. Furthermore, atRA treatment of AML cells demonstrates that m^6^A-related pathways can also be targeted as a potential strategy to enhance other available therapies [72]. Because m^6^A plays an important role in the biology of several viruses with considerable impact on human health, manipulating m^6^A regulation has also been explored as a potential anti-viral therapy. Use of 3-deazaadenosine (DAA), a drug that can block methylation, can inhibit replication of HIV-1 as well as RSV and influenza A virus [93], [108], [109], [110], [111]. Although these experiments do not conclusively show that m^6^A inhibition can be used as an antiviral therapy, since this drug is not an m^6^A specific inhibitor, several studies have demonstrated that targeting m^6^A pathway is a viable strategy. As m^6^A regulation is involved in multiple pathways, targeting m^6^A-dependent pathways can potentially lead to unwanted side effects. Therefore, development of drugs that inhibit these enzymes in a context-specific manner, or target the interaction between specific m^6^A targets and the interacting proteins will be critical for the success of m^6^A-targeted therapies.

## Competing interests

The author declares no competing interests.
